# Editorial: Innovative dental biomaterials for advancing oral health care

**DOI:** 10.3389/fdmed.2025.1643992

**Published:** 2025-07-03

**Authors:** Abdulrahman A. Balhaddad, Mary Anne S. Melo, Zuhair S. Natto

**Affiliations:** ^1^ Department of Restorative Dental Sciences, College of Dentistry, Imam Abdulrahman Bin Faisal University, Dammam, Saudi Arabia; ^2^ Department of Comprehensive Dentistry, University of Maryland School of Dentistry, Baltimore, MD, United States; ^3^ Department of Dental Public Health, Faculty of Dentistry, King Abdulaziz University, Jeddah, Saudi Arabia

**Keywords:** antimicorbials, bioactive, tissue regenartion, remineralization, biofilm, caries, periodontiti

**Editorial on the Research Topic**
Innovative dental biomaterials for advancing oral health care

## Introduction

The complexity of the oral environment poses a challenge to the clinical longevity of dental materials (1). These challenges involve several aspects related to the mechanical and biological performance of these materials. Dental materials inside the oral cavity are subjected to repetitive cycles of stress and fatigue. This mechanical challenge is complicated by the frequent exposure to consumable drinks and salivary enzymes, which may accelerate the degradation process of such materials (2). In addition, the interaction between dental materials and oral biofilms is a complex and dynamic process that can have significant implications for oral health (3). Dental materials provide a surface for the attachment and growth of oral bacteria. The attached microbes can produce acids as metabolic byproducts, leading to the degradation of dental materials (4). Besides, dental materials used in tissue regeneration and engineering are facing several complications related to achieving optimum vascularization, finding suitable scaffolds, cell differential, and immune response (5).

Such challenges have guided dental researchers to investigate advanced approaches to improve dental materials' mechanical and biological behavior. Applying nanotechnology in the dental field allows the engineering of dental materials with enhanced mechanical and physical properties (6). Additionally, incorporating bioactive compounds into dental materials contributes to the remineralization of tooth structure and the preservation of surrounding soft tissues by releasing ions or therapeutic compounds (7). The design of advanced dental materials with improved properties allows dental professionals to achieve superior treatment outcomes, enhance patient satisfaction, and provide more efficient and effective dental care. This Research Topic focuses on original research papers and comprehensive reviews that delve into pioneering advancements in dental materials.

### The Use of bioactive and reinforcing compounds in dental materials

Several articles discussed the incorporation of different compounds into dental materials to improve their antimicrobial action, biocompatibility, or mechanical performance. The impact of adding quaternary ammonium to endodontic sealers was explored in one of the studies. Alharamlah et al. incorporated dimethylaminohexadecyl Methacrylates (DMAHDM), as a contact-killing agent, into two different endodontic sealers, AH Plus and BC sealers. They found that 5 wt.% of DMAHDM in both sealers significantly inhibited the growth of *Enterococcus faecalis* biofilms over the sealers. This was achieved with no major adverse effects related to the flow, film thickness, contact angle, and solubility of the modified sealers in comparison to the control. Such findings may suggest that DMAHDM could be a promising agent to minimize the incidence of root canal reinfection when it is incorporated into endodontic sealers.

In another study, the use of a mussel-inspired polymerizable monomer [catechol–Lys–methacrylate (CLM)] as a primer to improve bonding to caries-affected dentin was investigated by Hu et al. They found that using CLM as a primed improved the micro-tensile bond strength to caries-affected dentin by almost 30%. Following thermocycling, the bonding strength was reduced by almost 41%, which was significant compared to a 13% reduction when the CLM primer was used. The CLM primer was also effective in minimizing the micro-leakage and imparting antibacterial properties in the bonding interface. The amount of *Streptococcus mutans* biofilm growth was reduced by 5-log when the CLM primed was used. In addition, the nano-leakage was reduced by 23% compared to the control.

To improve periodontal regeneration, Zhang et al. designed bone morphogenetic protein 2 (BMP2) peptide-modified polycaprolactone-collagen nanosheets (BPCNs) to enhance cell adhesion and osteogenesis. BPCNs exhibited remarkable biocompatibility, facilitating the adhesion of fibroblasts and bone marrow stem cells while also enhancing the osteogenic differentiation of bone marrow mesenchymal stem cells (BMSCs). Additionally, BPCNs substantially supported the regeneration of periodontal tissue in a rat model. Mechanistic insights from RNA sequencing analysis indicated that BPCNs led to the upregulation of genes associated with pro-inflammatory pathways.

Fouda et al. investigated the impact of nanoparticle addition and post-curing time on the mechanical performance of two types of rapidly prototyped (RP) denture-base resin (ASIGA vs. NextDent). The flexural strength improved with increased post-curing duration, reaching peak values for all tested groups at 90 min. Additionally, both materials exhibited a significant increase in flexural strength with the incorporation of nanodiamonds and silicon dioxide nanoparticles compared to the parental groups.

AlGhamdi et al. investigated the addition of 1 and 2 wt.% of titanium dioxide on the color stability and surface topographies of 3D-printed denture base resin (ASIGA and NextDent). In the case of NextDent, the addition of titanium dioxide led to a substantial color shift that surpassed acceptable limits and increased the roughness of the surface. However, no adverse effects were reported in relation to its hardness. Conversely, ASIGA's color alteration remained within clinically acceptable ranges. The surface roughness for ASIGA showed no change, while its hardness diminished with 2 wt.% of titanium dioxides.

In another article that aimed to explore a substitute for alcoholic mouthwashes, Alshehri et al. investigated the impact of *Caralluma munbyana* extracts on *S. mutans* biofilm growth. *C. munbyana* in its methanol and ethanol extracts significantly inhibited the *S. mutans* biofilms at the concentration of 23.44 mg/ml and higher, while the water extract was associated with minimum inhibition.

### The use of innovative approaches or techniques to improve the performance of dental materials

Several articles in this special issue investigated the impact of certain techniques or different parameters on the performance of dental materials. The first article published on the research topic was related to the effects of various surface treatments on the shear bond strength of clear aligner attachments bonded to Bis-acryl provisional crowns. Shahin et al. included five experimental surface treatments in their study, which were super coarse grit diamond bur, carbide bur, alumina-blasting, non-thermal plasma treatment, and erbium-doped yttrium aluminium garnet (Er:YAG) laser treatment. A group with no surface treatment was used as a control. They found that Plasma (10.69 ± 3.56 MPa), Er:YAG laser (9.68 ± 2.03 MPa), and alumina-blasting (9.15 ± 3.29 MPa) treatments yielded significantly higher shear bond strength compared to the control group (*p* < 0.05). Opposingly, Carbide and Diamond Bur (7.85 ± 2.1 MPa and 7.43 ± 3.3 MPa) did not illustrate any significant differences in SBS compared to the control (*p* > 0.05), with the lowest shear bond strength values recorded. Following the thermos-cycling challenge, all the groups demonstrated a significant decrease in shear bond strength, except for the alumina-blasted and plasma groups. The study concluded that the shear bond strength of aligner composite attachments was significantly enhanced by surface treatment using alumina-blasting, Er:YAG laser, and non-thermal plasma.

A similar study was conducted by Alzaid et al., but instead of clear aligner attachments, metal brackets were used and attached to 3D and milled provisional crowns. The crowns were treated either with 9.5% hydrofluoric acid or 37% of phosphoric acid prior to attaching the brackets. After brackets attachment, the crowns were subjected to thermocycling. In the context of 3D-printed materials, the results indicated that hydrofluoric acid etching produced a notably greater bond strength (12.59 ± 2.64 MPa) compared to phosphoric acid etching, which had a bond strength of 7.77 ± 0.83 MPa. Conversely, when examining milled materials, the bond strength values were lower, with hydrofluoric acid achieving 5.98 ± 0.59 MPa and phosphoric acid slightly lower at 5.66 ± 0.65 MPa, with no significant difference between the two etching materials.

A study by Al-Dulaijan et al. investigated the influence of different printing parameters on the internal and marginal fit of two provisional 3D-printed fixed partial dentures (ASIGA vs. NextDent). The study found that both printing orientation and post-curing time significantly affect the internal and marginal fit of the 3D-printed provisional prostheses. NextDent resin consistently provided a better overall fit than ASIGA resin. In ASIGA, a 0-degree printing orientation showed superior internal fit compared to 45- and 90-degree orientations. In NextDent, a 45-degree orientation improved internal fit. For marginal fit, ASIGA crowns performed best at a 90-degree orientation, while NextDent crowns thrived at a 45-degree orientation. Additionally, a longer post-curing time of 120 min benefited ASIGA resin, while 30 min was ideal for NextDent resin.

Another study by Al-Zain et al. investigated whether the placing of a second layer of universal adhesive with or without curing could improve the micro-tensile bond strength of resin-based composites. They found that the application of another layer of adhesive, either with or without curing, did not increase the bonding strength of the restoration.

### Mechanical and physical evaluation of new dental materials

The evolution of nanotechnology and industries introduce several dental materials to the market with no reported findings regarding their mechanical and physical evaluation. The special issue contains two articles that evaluated the performance of newly introduced dental materials in the market. Highly filled flowable resin-based composites have been used in a new technique to restore the anterior teeth, called the injectable technique. In Farghal et al. study, the staining susceptibility of injectable resin-based composites (Beautifil Flow Plus X and G-ænial Universal Injectable) was investigated. They found that injectable resin-based composites showed a reduced susceptibility to staining compared to their control counterparts. When different staining removal methods were investigated to see if the color change could be hindered, none of the methods used for removing stains were able to fully restore the original color of any of the investigated materials.

Farghal et al. also conducted another article to investigate the effect of carbonated beverages on the color and microhardness of bulk-fill resin-based composites (BEAUTIFIL-Bulk Restorative and Filtek One Bulk-fill) with and without preheating. The preheating process of bulk-fill resin-based composites notably enhanced their microhardness and elevated the surface gloss of the Filtek One Bulk-fill. However, preheating did not provide adequate protection for either composite against the acidic effects of Cola drinks. While the preheated Filtek One Bulk-fill exhibited better color stability following immersion in Cola, the improvement was still insufficient to meet clinically acceptable standards.

### Review articles

This special issue contains a systematic review and meta-analysis that investigates the role of silver diamine fluoride (SDF) in caries prevention and arrest. Alqalaleef et al. analyzed 20 studies and reported that, in both pediatric and adult patients, the rate of caries arrest using SDF ranged from 25% to 99%. While such findings reveal the effectiveness of SDF in caries prevention, it is important to highlight that its efficiency was almost comparable to other caries prevention approaches.

In a review article written by Levina and Dubnika, combining platelet-rich fibrin (PRF) with other biomaterials for improved tissue regeneration was explored. Different biomaterials, such as nano-hydroxyapatite, calcium phosphate compounds, and bone substitute materials, exhibit various properties related to bone formation and healing time. It was concluded that the combination of PRF with other biomaterials appears to hold considerable potential for improving bone regeneration and healing. However, it was highlighted in the review that the results explored are inconsistent, necessitating the need for standardized protocols and conducting more clinical trials.

Alluhaidan et al. in their scoping review article explore the different dental applications of quantum dots. Most of the research (82%) concentrated on treatment applications of quantum dots in dentistry, whereas a smaller segment (18%) explored their use in imaging technologies. Most of the quantum dots investigated were graphene-, metal oxide-, and carbon-based, with hydrothermal being the most common method for synthesis. The incorporation of quantum dots in different dental technologies was mainly to impart antimicrobial and remineralization properties, enhance bone regeneration, and improve fluorescence activities. The review highlights the need for long-term studies to validate the benefits of using quantum dots in dentistry.

## Conclusion

The collection of articles in this special issue highlights the necessity of exploring additional bioactive compounds and innovative techniques, along with their applications in dentistry. This exploration can enhance oral healthcare by improving the performance of dental materials and maximizing their clinical benefits. The topic editors wish to stress the importance of using clinical translational models to evaluate dental materials in accordance with their clinical applications. Many innovative materials that demonstrate promising performance *in vitro* may encounter technical or biological challenges when applied *in vivo*. Therefore, adapting clinical models to test these materials and techniques is essential for validating their clinical benefits.
